# Signal transduction, receptors, mediators and genes: younger than ever - the 13th meeting of the Signal Transduction Society focused on aging and immunology

**DOI:** 10.1186/1478-811X-8-2

**Published:** 2010-02-11

**Authors:** Frank Entschladen, Joachim Altschmied, Ria Baumgrass, Iris Behrmann, Klaudia Giehl, Heike Hermanns, Otmar Huber, Arnd Kieser, Lars-Oliver Klotz, Katharina F Kubatzky, Ralf Hass, Ottmar Janssen, Karlheinz Friedrich

**Affiliations:** 1 Institute of Immunology, Witten/Herdecke University, Germany; 2 Environmental Health Research Institute at the Heinrich-Heine-University, Düsseldorf, Germany; 3 German Rheumatism Research Center, Berlin, Germany; 4 Life Science Research Unit, University of Luxembourg, Luxembourg; 5 Internal Medicine I, University Hospital Ulm, University of Ulm, Germany; 6 Rudolf-Virchow-Center, DFG-Research Center for Experimental Biomedicine, University of Würzburg, Germany; 7 Department of Biochemistry II, Jena University Hospital, Jena, Germany; 8 Department of Gene Vectors, Helmholtz Center Munich - German Research Center for Environmental Health, Munich, Germany; 9 Medical Microbiology and Hygiene, Ruprecht-Karls-University, Heidelberg, Germany; 10 Department of Gynecology, Laboratory of Biochemistry and Tumor Biology, Medical University of Hannover, Germany; 11 Molecular Immunology, Institute of Immunology, Christian-Albrechts-University, Kiel, Germany

## Abstract

The 13th meeting of the Signal Transduction Society was held in Weimar, from October 28 to 30, 2009. Special focus of the 2009 conference was "Aging and Senescence", which was co-organized by the SFB 728 "Environmentally-Induced Aging Processes" of the University of Düsseldorf and the study group 'Signal Transduction' of the German Society for Cell Biology (DGZ). In addition, several other areas of signal transduction research were covered and supported by different consortia associated with the Signal Transduction Society including the long-term associated study groups of the German Society for Immunology and the Society for Biochemistry and Molecular Biology, and for instance the SFB/Transregio 52 "Transcriptional Programming of Individual T Cell Subsets" located in Würzburg, Mainz and Berlin. The different research areas that were introduced by outstanding keynote speakers attracted more than 250 scientists, showing the timeliness and relevance of the interdisciplinary concept and exchange of knowledge during the three days of the scientific program. This report gives an overview of the presentations of the conference.

## 

The scientific program of the 13th annual conference of the Signal Transduction Society started with a workshop on Immune Signaling that was co-organized by the study group 'Signal Transduction" of the German Society for Immunology and The SFB/Transregio 52 "Transcriptional Programming of Individual T Cell Subsets" with scientists from Würzburg, Mainz and Berlin. The introductory keynote lecture was given by Arthur Weiss from the Howard Hughes Medical Institute in San Francisco, CA, the past president of the American Association of Immunology (AAI) and an outstanding pioneer of T cell signal transduction research. Professor Weiss discussed the requirements and properties of inhibitors of protein tyrosine kinases for their clinical use. In particular, he reported that certain inhibitors induce quite dramatic and relevant conformational changes in the key tyrosine kinase ZAP-70 that alter the accessibility of the molecule and regulate the state of activation be fine-tuning intra- and intermolecular protein-protein interaction.

Following the STS tradition, the workshop was continued by short presentations selected from the more than 120 submitted abstracts. Dirk Mielenz (University of Erlangen-Nuremberg) introduced Swiprosin-1/EFhd2, a scaffold protein that for example enables the recruitment of the Syk kinase and other signal transducers such as PLCγ2 to B cell receptor associated lipid raft areas.

Sonja Reißig (University Hospital Mainz) discussed the function of the tumor suppressor gene CYLD in T cell development and NF-κB mediated signaling of T cells highlighting that the expression of a short splice variant CYLD^ex7/8 ^in mice leads to reduced numbers of CD8^+ ^and CD4^+ ^T lymphocytes in the thymus and the periphery and thereby indicating that CYLD seems to be relevant for the developing T cell compartment.

The role of the Nuclear Factor of Activated T cells c1 (NFATc1) in B cell receptor signaling was analyzed by Jolly Deb (University of Würzburg) who demonstrated that an inactivation of NFATc1 leads to increased activation induced cell death (AICD) also in the B cell compartment.

The second part of the Immune Signaling Workshop started with a keynote presentation given by Anjana Rao from the Dana-Farber Cancer Institute at Harvard Medical School, Boston. She reported novel insights into the function of Foxp3 related to its binding properties as well as suppressive abilities. The DNA-binding sites in the forkhead domain of Foxp2 differ from those of Foxp3 in three amino acids. If these variant residues are introduced into Foxp3 and the resulting mutant protein is over-expressed in cell lines, the suppressive activity but not DNA binding of Foxp3 is abrogated. Moreover, these residues are important for Foxp3 dimer formation that bridges two molecules of DNA. Introduction of structure-guided mutations in the domain-swapping interface of Foxp3 specifically disrupted this dimer formation and impaired the suppressor function on retrovirally-transduced T cells but compromised the ability of Foxp3 to bind to DNA. Further evaluation will show whether the Foxp3-mediated long-range interactions in DNA may contribute in part to the gene regulation by Foxp3 in regulatory T cells.

Related to this topic, Matthias Klein and colleagues (University of Mainz) discussed the role of the Ikaros transcription factor Helios in regulatory T (Treg) cells. Helios is highly expressed in Treg cells and is not a target of Foxp3 but rather promotes Foxp3 expression. Thus, Helios may play a role in Treg cell induction and function.

Thomas Oellrich and colleagues (University of Göttingen) demonstrated the great potential of a functional proteomic approach to characterize the basal state and the kinetics of signaling modules after signal processing. They elucidated the functional interactomes of the adapter protein SLP-65 and its regulation by BCR signaling. They combined three techniques: identification of molecules in signaling complexes by stable isotope labeling with amino acids in cell culture (SILAC), analysis of the affinity-purified molecules by mass spectrometry, and evaluation of the functionality of the molecules by mutational reconstitution studies. This approach seems to be promising in studying checkpoints of BCR and TCR signaling in general.

Markus Lettau and colleagues (University of Kiel) have been interested in the identification of interaction partners for the intracellular N-terminal region of Fas ligand that might be generated by proteolytic shedding during T cell activation. Using the phage display technique, they identified several potential binding proteins including tyrosine kinases and different families of adaptor proteins including PCH proteins and sorting nexins. The differential binding of individual interactors to the full length Fas ligand protein and/or the N-terminal fragments might indicate a regulatory capacity of the proteolytically processed FasL N-terminus.

The workshop Growth Factors, Cytokines and Chemokines started with a presentation of the keynote speaker Ivo Touw (Erasmus University Medical School Rotterdam) about the granulocyte colony stimulating factor receptor (GCSFR). For this receptor, a tight interplay between receptor routing and signal transduction could be demonstrated: Upon GCSF stimulation and endocytosis, the receptor becomes ubiquitinylated at lysine 632 in a SOCS3-dependent manner. This ubiquitinylation is responsible for the lysosomal targeting and degradation of the receptor. Mutation of lysine 632 leads to prolonged activation of STAT5 and Erk (as long as there is no other, substituting lysine residue present in the juxta-membrane region), but not of protein kinase B. Novel data show that GCSF quickly leads to the expression of a deubiquitinylating enzyme (DUB2A) which can counteract the SOCS3-mediated ubiquitinylation and thereby stabilize the GCSFR in early endosomes. Moreover, GCSF signaling is affected by radical oxygen species. Most interestingly, the C-terminal part of the GCSFR can interact with an ER-resident peroxiredoxin (PRDX4). According to the current working model, the internalized receptor transits the ER where PRDX4 may act to prevent the inactivation of redox-sensitive phosphatases and thereby favor dephosphorylation events leading to the termination of signal transduction.

Heike M. Hermanns (Rudolf-Virchow-Center, University of Würzburg) gave a short presentation about the significant involvement of Oncostatin M (OSM) in rheumatoid arthritis. OSM, but not IL-6 or TNFα, can stimulate the expression of the chemokine CCL13 in synovial fibroblasts, but not in other types of fibroblasts or chondrocytes. STAT5, Erk1/2 and p38 are involved in the OSM-dependent transcription and mRNA stabilization of CCL13. CCL13 was discovered in the synovial fluid of patients with rheumatoid arthritis (RA) and stimulates the migration of monocytes. Interestingly, synovial fibroblasts of RA patients constitutively produce CCL13, which is partially dependent on endogenous OSM production.

Ignacio Rubio (University of Jena) developed a novel high avidity probe for RasGTP which can be used to monitor Ras activity as well as its subcellular distribution. It is composed of three copies of GFP and three copies of the Ras binding domain of Raf. In contrast to previously developed Ras probes, this novel construct is able to decorate endogenous RasGTP. Using the novel probe to image Ras activation by the T cell receptor, Rubio and coworkers identified the plasma membrane as the sole site of Ras activation in life T-cells.

TAK1, an important upstream kinase in the signaling pathways involving IL-1, TNF, or toll-like receptors, is controlled by the regulatory subunit TAB1. Alexander Wolf (University of Giessen) reported novel phosphorylation sites within the C-terminal cluster of six serine residues of TAB1. The phosphorylation can be mediated by TAK1 as well as by p38 MAPK and seems to affect the activity and localization of p38 MAPK.

The data presented by Katharina Rzeczkowski (University of Giessen) point at an exciting novel role of JNK in post-transcriptional events: argonaute 2 (Ago2), the essential catalytic subunit of the RISC complex, is a binding partner of JNK. A potential role of JNK in miRNA regulation is implied by an altered miRNA expression in JNK-deficient cells and by an altered binding pattern of certain miRNAs in Ago2/JNK complexes upon JNK phosphorylation. Moreover, Ago2 and JNK colocalize in cytoplasmic processing (P)-bodies together with the decapping complex subunit DCP1α which was further demonstrated to be a JNK substrate. JNK activity affected the number and size of DCP1α-containing RNP particles. These data suggest that JNK participates in the regulation of mRNA processing.

Among the six short presentations of the second part of the workshop on Growth Factors, Cytokines and Chemokines two talks addressed novel aspects of CD95/Fas mediated signaling. Inna Lavrik from the group of Peter Krammer (Division of Immunogenetics, DKFZ Heidelberg) used a systems biology approach to understand the life and death decisions mediated by CD95 (Fas, APO-1) in quantitative terms. Using HeLa cells which overexpress various components of the CD95 system they showed both apoptotic and non-apoptotic pathways depending on the concentration of the CD95 death-inducing signaling complex (DISC) components. Single-cell and population-based measurements resulted in a mathematical model which takes into account the complex interplay between various death effector domain (DED)-containing proteins. The application of this model has demonstrated how exact concentrations of c-FLIP and procaspase-8 and their cleavage products at the DISC can determine life/death decisions.

Ottmar Janssen (University of Kiel) highlighted the dose-dependent modulation of CD4 T-cell responses by CD95 co-stimulation. They confirmed that CD95 can exert anti-apoptotic activities under certain conditions. High doses of CD95 agonists completely abrogated CD3/CD28-triggered T-cell proliferation without inducing cell death. Early as well as late signaling events were equally impaired. In contrast, lower doses drastically augmented CD3-induced cell proliferation which was due to co-internalization of the receptors and sustained activation of signaling components (MAP kinases, caspases, NF-κB and cell cycle components) and resulted in upregulation of various activation markers (adhesion molecules, cytokine receptors, cytokines).

The group of Stefanie Kliche (University of Magdeburg) investigated the role of the adapter protein SLP76 (SH2 domain containing leukocyte-specific phosphoprotein of 76 kDa) in T-cell receptor (TCR) and chemokine receptor (CXCR4) signaling, both of which are crucial for adhesion and migration of T-cells. They identified the mandatory function of SLP76 in TCR-mediated inside-out signaling, where it facilitates the membrane recruitment of the ADAP/SKAP55/RIAM/Rap1 module and controls the Rac-mediated actin polymerization. Surprisingly, CXCR4-mediated T-cell adhesion and migration was not affected by the loss of SLP76 implying a differential role for this adapter protein in TCR- and CXCR4-mediated inside-out signaling.

Yaw Asare from the group of Jürgen Bernhagen (RWTH Aachen) demonstrated a novel role of the COP9 signalosome (CSN) in NF-κB signaling in atherogenesis. Silencing CSN5/JAB1, a critical subunit of the COP9 complex and responsible for the cullin-1 deNEDDylation activity of CSN, resulted in an enhanced expression of the inhibitor of κB (IκBα). In the context of inflammatory or pro-atherogenic conditions CSN5 or CSN2 knockdown increased the TNFα-mediated phosphorylation of IKK and NF-κB which might be indicative of a vital role of CSN in the canonical NF-κB pathway.

Meike Jung also from the group of Jürgen Bernhagen addressed the signaling and function of the cytokine MIF (macrophage migration inhibitory factor) in liver fibrogenesis. To this end they compared *mif*^-/-^with wild-type mice in the CCl_4_- and the thioacetamide-induced fibrosis model. Immunohistochemical analysis as well as evaluation of fibrosis markers revealed a stronger fibrotic response in *mif*^-/-^mice. This suggested an unexpected protective role of MIF during the progression of liver fibrosis.

Finally, the group of Iris Behrmann (Life Sciences Research Unit, University of Luxemburg) identified a novel physiological function of the interleukin-6-type cytokine oncostatin M. In hepatocytes and hepatoma cells this cytokine upregulated the expression of hypoxia-inducible factor-1α (HIF-1α). This protein is most often regulated on the level of protein stability; however, here it was clearly shown that increased expression of HIF-1α in response to OSM did not result from protein stabilization but from increased transcription. Behrmann *et al*. identified the Jak/STAT3- and MEK/Erk1/2-pathways as crucial signaling components important for OSM-induced HIF-1α transcription and consequently for the enhanced expression of VEGF and PAI-1. Their results therefore point at a crosstalk between cytokine and hypoxia signaling in liver development or regeneration.

The workshop Migration, Adhesion and Intercellular Signalling started with a keynote lecture by Stephen Ward (University of Bath, UK), who highlighted the essential role of phosphytatidylinositol-3-kinases (PI3K) in the polarization and migration of leukocytes in inflammation and autoimmune diseases. He discussed the potential use of PI3K inhibitors, and showed that alternatively an activation of the antagonistic phosphatase SHIP (SH2-containing inositol-5-phosphatase) might provide a target of hematopoietic and immune disorders in which the PI3K pathway is dysregulated.

The targets of phosphoinositides were the topic of Kerstin Tang's (Charité, Berlin) presentation. She provided evidence that the α1β1 integrin, a receptor for collagens and laminin1, which is important for cell migration, interacts with phosphoinositides, especially with phoaphatidylinositol 4,5-bisphosphate and phosphatidylinositol 3,4,5 trisphosphate, via its positively charged cytoplasmic which consists of only 15 amino acids. Binding of the integrin to the negatively charged phosphoinositides of the plasma membrane might be an early event, which is important for the regulation of integrin affinity and subsequently, for the modulation of cell migration.

Michal Smida (University of Magdeburg) presented results on the identification of a novel GTPase-activating protein, which they termed IGAP (inducible GTPase-activating protein), since this protein is upregulated upon T cell activation. The targets of IGAP are the GTPases Rac and Cdc42, but not Ras and Rap1. He proposed that the upregulation of IGAP in activated T cells serves to regulate their activation status.

Thomas Vogl (University of Münster) described the function of the calcium-binding protein complex S100A8/S100A9 in the migration of leukocytes. He discussed data on the interaction of this complex with microtubules and the regulation of the dynamic behavior of microtubules. The S100A8/S100A9 complex is also known as calprotectin, which is secreted by phagocytes during stress response as an endogenous activator of the toll-like receptor 4.

The scientific part of the session on Migration, Adhesion and Intercellular Signalling closed with a presentation by Otmar Huber (University of Jena) elucidating that the transcriptonal activity of the estrogen receptor (ER) is enhanced by β-catenin. He described the canonical Wnt-signaling and further modulators of ER activity in anchorage-dependent growth of cancer cells.

Bacterial and viral strategies to modulate host cell signaling are a constantly expanding field of research and were the main topic of the workshop on Pathogens and Disease. Pathogenic microbes and viruses exploit cellular signaling pathways to establish a successful infection and to block or evade the host's defense mechanisms against the invader. In his keynote presentation, Michael Naumann (University of Magdeburg) reviewed current knowledge on immunomodulatory functions of pathogens, exemplified by bacterial and viral mechanisms targeting the NF-κB pathway, a crucial coordinator of the innate and adaptive immune response. Many components of the NF-κB pathway are regulated at the post-transcriptional level by secondary modifications through ubiquitin (Ub) or ubiquitin-like modifiers (Ubl) such as Nedd8 or SUMO. Although bacteria themselves do not possess an Ub or Ubl system, it was found recently that bacterial pathogens express deubiquitinating enzymes (DUBs) to modulate the NF-κB system of the host, which is a pivotal example of co-evolution of the host and its pathogens. For instance, *Chlamydia trachomatis*, a human pathogen that causes various human diseases including blindness, evades the host inflammatory response by expressing the deubiquitinating ChlaDub1 protein that inhibits I-κB ubiquitination and, thus, NF-κB activation. Modulation of the host's Ub-system likely constitutes a general strategy used by many pathogens because some viruses encode DUBs as well. A newly described example is the Kaposi's sarcoma-associated herpesvirus (KSHV) Orf64 DUB, which possesses target specificity towards Lys 48 or Lys 63-linked ubiquitin chains.

Dagmar Hildebrand and colleagues (University of Heidelberg) presented data on the mitogenic protein toxin PMT from toxigenic *Pasteurella multocida *strains. After their initial observation that PMT is capable of blocking the apoptotic effects of the omnipotent kinase inhibitor staurosporine, Dagmar Hildebrand and co-workers investigated the mechanism of this signaling pathway. They found that two serine/threonine kinases are involved, as PMT leads to STAT-dependent induction of Pim-1 expression and PI3K-dependent Akt activation. Selective inhibition of both kinases was necessary and sufficient to re-sensitise cells for staurosporine.

Mauro Togni (University of Magdeburg) and colleagues investigated the mechanisms enabling immune cells to eliminate intracellular pathogens such as *Listeria monocytogenes*. They found that mice deficient for the cytosolic adapter molecule SKAP-HOM have a reduced potential to clear the pathogen. Subsequently, they identified a role of SKAP-HOM in signal transduction of dendritic cells (DC). Although SKAP-HOM-deficient DCs were able to transduce toll-like receptor (TLR)-mediated signals, these cells showed reduced actin polymerization, downregulation of proteins involved in cytoskeletal rearrangement, such as cofilin or Pyk2, and an upregulation of pSTAT5 levels upon pathogen stimulation. These changes eventually caused a reduced number of CD8^+ ^T cells and incomplete pathogen clearance *in vivo*. The data from Togni et al. therefore suggest that not only pathogen recognition through TLRs but also intercellular signaling via integrins plays an important role in the efficient activation of the adaptive immune system.

Signalling mechanisms of herpesviral oncoproteins were the focus of the two talks on viral pathogens. Anna Shkoda and colleagues (Helmholtz Center Munich) reported a novel experimental strategy by which they were able to identify new components of the signaling complex of the latent membrane protein 1 (LMP1) of Epstein-Barr virus (EBV) in EBV-transformed primary human B-lymphocytes. Using subtractive proteomics they have characterized a member of the STE20 family of germinal center kinases with so far largely unknown functions in hematopoietic cells as a novel interaction partner of the LMP1 complex. Data were presented demonstrating that interaction of this kinase with the very C-terminus of the LMP1 signaling domain is mediated by TRAF6, a critical signaling mediator of LMP1 and cellular members of the TNF-receptor and TLR families. RNAi experiments finally proved that this STE20 kinase is essential for LMP1 activation of the cellular JNK pathway, which is required for host cell transformation by LMP1.

In the last presentation of the session on Pathogens and Disease, Stefan Richter (University Hospital Erlangen) reported on a newly identified cross-talk of the herpesvirus saimiri (HVS) oncoprotein Tip (tyrosine kinase interacting protein) with death receptor signaling in Jurkat cells as well as in HVS-transformed human T-cells. Tip activates pro-apoptotic caspases via a Fas- and TRAIL-independent mechanism. Notably, Tip seems to subvert and, thereby, exploit death receptor signaling pathways for cell transformation because Tip-expressing cells were protected from apoptosis. The precise underlying molecular mechanism of this effect is currently under investigation.

The special focus topic of the 2009 meeting of the Signal Transduction Society was Aging. The first of the two workshops on Aging was devoted to Mechanisms and Biomarkers of Aging. Two keynote presentations highlighted the two foci set by the title of the session: Alexander Bürkle (University of Konstanz) introduced a novel project funded by the EU commission, MARK-AGE, that is designed as a population study serving the purpose of identifying biomarkers of aging that may be applied as a set of parameters in order to estimate an individual's biological age. One of the goals is to enhance the predictability of the onset of age-related diseases.

Jose Viña (University of Valencia, Spain) presented data on the identification of novel longevity-associated genes, including p53/p16 and guanosine releasing factor 1 (GRF1). While the presence and activity of p53 was demonstrated to enhance longevity by upregulating the expression of potent antioxidants, the sestrins, life span is enhanced by the loss of GRF1, which is accompanied by a downregulation of IGF1. Current research aims at identifying physiological approaches inducing changes in expression of longevity-associated genes.

Age-related damage to biomolecules can serve as potential biomarker for aging and may also be causative in bringing about the very changes observed in cellular metabolism and tissue integrity during senescence. Two variations on that theme were presented by Andreas Simm (University of Halle-Wittenberg) and Rudolf Wiesner (University of Cologne): The accumulation of advanced glycation endproducts not only serves as a potential biomarker of aging but glycation of platelet-derived growth factor (PDGF) was demonstrated by Simm et al. to impair PDGF signaling, with potential implications for physiological processes such as wound healing. Wiesner et al., on the other hand, demonstrated that damage to a different type of biomolecule, mitochondrial DNA (mtDNA), not only accumulates during aging and in age-related pathologies, but may be enhanced by the turnover and metabolism of well-known signaling molecules, catecholamines. Accumulation of damage to mtDNA will result in impaired mitochondrial metabolism and activity that may, in turn, foster the development of age-related changes in tissues.

The second part of the special focus theme Aging had the topic DNA Damage Repair and Aging. In the keynote lecture, Ian D Hickson (University of Oxford, UK) introduced the ReqQ helicase family, which is required for the maintenance of genomic stability and thus serves important functions in the prevention of cancer. The role of these proteins in aging was underscored by the fact that a compromised function of different RecQ helicases in several human genetic disorders does not only lead to cancer predisposition, but also to various progerias, among them Bloom's and Werner's syndrome.

The talk by Marc Majora (IUF, Düsseldorf) described an interesting phenotype of mice deficient in the CSB protein, which is part of the transcription-coupled repair (TCR) pathway. The knockout of CSB led to increased papilloma formation, but unexpectedly resulted in protection against UV-induced carcinogenesis. This indicates opposite functions of TCR in photoaging and photocarcinogenesis in the skin.

Cornelia Dietrich (University Medical Center, Mainz) described beneficiary roles of dietary ligands of the aryl hydrocarbon receptor (AhR), which is known to mediate toxic effects of many environmental pollutants, e.g. dioxin. A metabolic product of brassica vegetables was shown to protect against oxidative DNA damage.

The remaining talks described different experimental approaches to analyze the processes underlying cellular and organismic aging ranging from biochemical and cell culture studies to in vivo analyses in different animal model systems. Susanne Jennek, (Jena University Hospital) presented a proteomic approach directed at identifying interaction partners of the p33^ING1B ^protein, which can induce premature senescence in primary human fibroblasts.

Under the aspect that mitochondrial dysfunction is observed during aging, Kim Zarse (University of Jena) showed in *Caenorhabditis elegans *that a transient increase in mitochondrial ROS formation can potentiate the life span extension observed when the Insulin/IGF-I signaling pathway is impaired.

Nicole Büchner (IUF, Düsseldorf) described non-nuclear functions of the catalytic subunit of telomerase, telomerase reverse transcriptase (TERT). TERT was shown to be present in mitochondria, where it protected mitochondrial DNA against damage and improved mitochondrial function in cell culture and in vivo.

Michael Graf (Leibniz Institute for Age Research, Jena) introduced a new animal model system in aging research, the fastest aging vertebrate known thus far, the annual killifish *Nothobranchius furzeri*, which has been established as a laboratory animal only during the last few years.

This year's session on recent advances in Tumor Biology was introduced by Stefan Knapp (University of Oxford, UK) who gave an overview on new insights into kinase regulation and kinase inhibition resulting from high-throughput ultrastructural experiments. Many kinases have been linked to disease development and several of them are validated intervention points for inhibitor and drug development. However, clinical trials demonstrate that kinase inhibitors are often not as specific as desired leading to unwanted side effects. The group of Stefan Knapp described more than 50 novel catalytic domain structures and kinase inhibitor complexes and identified novel structural motifs that represent a valuable structural genomics platform for drug design and development. In his talk he also presented recent results on kinase regulation and auto-activation especially concentrating on calcium calmodulin-dependent kinases.

Christina Zechel (University Hospital of Schleswig-Holstein) focused in her talk on the responsiveness of primary cultures of stem cell-like brain tumor cells to mitogens. The presented data indicate that neural stem cell (NSC)-like cells within malignant brain tumors support the growth of tumor cells by paracrine mechanisms. Moreover, NSC-like cells themselves respond to mitogens, such as EGF, bFGF or PDGF.

The role of the transcription factor NFAT in T-cell acute lymphoblastic leukaemia (T-ALL) was introduced by Martin R. Müller (University of Tübingen). Experiments in NIH/3T3 mouse fibroblasts demonstrated that constitutive active NFAT1 and NFAT2 exhibit opposite effects on proliferation and cell growth. NFAT2 promotes proliferation and growth whereas NFAT1 suppresses cell growth and proliferation. By using a T-ALL transgenic mouse model expressing hyperactive NFAT1 in T cells the tumor suppressive function of NFAT1 was affirmed thereby emphasizing the importance of the NFAT signaling pathway in the pathogenesis of leukemia.

The regulation of a second family of transcription factors of special relevance in T lymphocytes, namely NF-κB, was addressed by Sarah Jill de Jong (University Erlangen-Nuremberg). The *Herpesvirus ateles *T lymphotropic oncoprotein "two-in-one" (Tio) is a membrane-bound protein which forms homodimers or multimers. Tio markedly induced NFκB in T cells by TRAF6- and Nemo-dependent pathways and it initiated the processing of active p52 independent of the canonical NF-κB signal. Thus, Tio might be a unique tool to study non-canonical NF-κB signaling in T cells.

The important topic of cancer cell invasiveness was addressed by Gerald Radziwill (University of Freiburg). The scaffold protein CNK1 mediates proliferative as well as antiproliferative responses and was described to be involved in protooncogenic signaling pathways. G. Radziwill demonstrated that CNK1 is localised at the plasma membrane in breast carcinoma cells where it supports cell proliferation in a phosphatdylinositiol-3-kinase (PI3K)-Akt-FoxO-dependent manner and induces tumor cell invasiveness in a NF-κB-dependent mode. Hence, CNK1 might be an essential mediator of oncogenic signaling in breast carcinoma cells and a new target in cancer therapy.

## Concluding remarks

The 2009 meeting of the Signal Transduction Society brought together a vivid community of scientists working on distinct aspects of signal transduction, but often benefiting a common basis of emerging tools and approaches, which enabled a productive exchange of new tricks of the trade and fostered many new and sometimes interdisciplinary collaborations. The 2010 conference will be held again in Weimar from 18 to 20 October. The special focus topic for 2010 will be "Drug Design/Drug Discovery".

## Competing interests

The authors declare that they have no competing interests.

## Authors' contributions

FE collected information from all other authors, organized and edited the contributions and drafted the manuscript. FE, JA, RB, IB, KG, HH, OH, AK, LOK, KFK, provided individual sections of text. FE, RH, OJ and KF are the council of the Signal Transduction Society and organizers of the conference. All authors read and approved the final manuscript.

